# Supplementary material for: Mobile app review analysis for crowdsourcing of software requirements: a mapping study of automated and semi-automated tools
Source: PeerJ Comput Sci. 2024 Nov 5;10:e2401. doi: 10.7717/peerj-cs.2401 (PMC11623114; doi:10.7717/peerj-cs.2401)
Supplement: Supplemental Information 1 [file peerj-cs-10-2401-s001.docx]

Appendix A

Quality Assessment Scores of Selected Primary Studies

| **Study ID** | **Q1** | **Q2** | **Q3** | **Q4** | **Q5** | **Q6** | **Q7** | **Q8** | **Q9** | **Q10** | **Total Score** |
| --- | --- | --- | --- | --- | --- | --- | --- | --- | --- | --- | --- |
| Study 1 | 1 | 1 | 1 | 0.5 | 1 | 1 | 0.5 | 1 | 1 | 1 | 9 |
| Study 2 | 1 | 0.5 | 1 | 1 | 0.5 | 0.5 | 1 | 1 | 0.5 | 1 | 8 |
| Study 3 | 1 | 1 | 0.5 | 0.5 | 1 | 1 | 0.5 | 0.5 | 1 | 1 | 8 |
| Study 4 | 1 | 0.5 | 1 | 1 | 1 | 0.5 | 1 | 1 | 1 | 0.5 | 8.5 |
| Study 5 | 0.5 | 1 | 1 | 0.5 | 0.5 | 1 | 0.5 | 1 | 0.5 | 1 | 7.5 |
| Study 6 | 1 | 0.5 | 0.5 | 1 | 1 | 0.5 | 1 | 0.5 | 1 | 1 | 8 |
| Study 7 | 1 | 1 | 1 | 0.5 | 0.5 | 1 | 0.5 | 1 | 0.5 | 0.5 | 7.5 |
| Study 8 | 0.5 | 0.5 | 1 | 1 | 1 | 0.5 | 1 | 1 | 1 | 1 | 8.5 |
| Study 9 | 1 | 1 | 0.5 | 0.5 | 0.5 | 1 | 0.5 | 0.5 | 1 | 1 | 7.5 |
| Study 10 | 1 | 0.5 | 1 | 1 | 1 | 1 | 1 | 1 | 1 | 0.5 | 9 |
| Study 11 | 0.5 | 1 | 1 | 0.5 | 0.5 | 0.5 | 0.5 | 1 | 0.5 | 1 | 7 |
| Study 12 | 1 | 0.5 | 0.5 | 1 | 1 | 1 | 1 | 0.5 | 1 | 1 | 8.5 |
| Study 13 | 1 | 1 | 1 | 0.5 | 0.5 | 0.5 | 0.5 | 1 | 0.5 | 0.5 | 7 |
| Study 14 | 0.5 | 0.5 | 1 | 1 | 1 | 1 | 1 | 1 | 1 | 1 | 9 |
| Study 15 | 1 | 1 | 0.5 | 0.5 | 0.5 | 0.5 | 0.5 | 0.5 | 0.5 | 1 | 6.5 |
| Study 16 | 1 | 0.5 | 1 | 1 | 1 | 1 | 1 | 1 | 1 | 0.5 | 9 |
| Study 17 | 0.5 | 1 | 1 | 0.5 | 0.5 | 0.5 | 0.5 | 1 | 0.5 | 1 | 7 |
| Study 18 | 1 | 0.5 | 0.5 | 1 | 1 | 1 | 1 | 0.5 | 1 | 1 | 8.5 |
| Study 19 | 1 | 1 | 1 | 0.5 | 0.5 | 0.5 | 0.5 | 1 | 0.5 | 0.5 | 7 |
| Study 20 | 0.5 | 0.5 | 1 | 1 | 1 | 1 | 1 | 1 | 1 | 1 | 9 |
| Study 21 | 1 | 1 | 0.5 | 0.5 | 1 | 0.5 | 0.5 | 0.5 | 1 | 1 | 7.5 |
| Study 22 | 0.5 | 0.5 | 1 | 1 | 0.5 | 1 | 1 | 1 | 0.5 | 0.5 | 7.5 |
| Study 23 | 1 | 1 | 1 | 0.5 | 1 | 0.5 | 0.5 | 1 | 1 | 1 | 8.5 |
| Study 24 | 0.5 | 0.5 | 0.5 | 1 | 1 | 1 | 1 | 0.5 | 1 | 1 | 8 |
| Study 25 | 1 | 1 | 1 | 0.5 | 0.5 | 0.5 | 0.5 | 1 | 0.5 | 0.5 | 7 |
| Study 26 | 1 | 0.5 | 1 | 1 | 1 | 1 | 1 | 1 | 1 | 1 | 9.5 |
| Study 27 | 0.5 | 1 | 0.5 | 0.5 | 0.5 | 0.5 | 0.5 | 0.5 | 0.5 | 1 | 6 |
| Study 28 | 1 | 0.5 | 1 | 1 | 1 | 1 | 1 | 1 | 1 | 0.5 | 9 |
| Study 29 | 0.5 | 1 | 1 | 0.5 | 0.5 | 0.5 | 0.5 | 1 | 0.5 | 1 | 7 |
| Study 30 | 1 | 0.5 | 0.5 | 1 | 1 | 1 | 1 | 0.5 | 1 | 1 | 8.5 |
| Study 31 | 1 | 1 | 1 | 0.5 | 0.5 | 0.5 | 0.5 | 1 | 0.5 | 0.5 | 7 |
| Study 32 | 0.5 | 0.5 | 1 | 1 | 1 | 1 | 1 | 1 | 1 | 1 | 9 |
| Study 33 | 1 | 1 | 0.5 | 0.5 | 0.5 | 0.5 | 0.5 | 0.5 | 0.5 | 1 | 6.5 |
| Study 34 | 1 | 0.5 | 1 | 1 | 1 | 1 | 1 | 1 | 1 | 0.5 | 9 |
| Study 35 | 0.5 | 1 | 1 | 0.5 | 0.5 | 0.5 | 0.5 | 1 | 0.5 | 1 | 7 |
| Study 36 | 1 | 0.5 | 0.5 | 1 | 1 | 1 | 1 | 0.5 | 1 | 1 | 8.5 |
| Study 37 | 1 | 1 | 1 | 0.5 | 0.5 | 0.5 | 0.5 | 1 | 0.5 | 0.5 | 7 |
| Study 38 | 0.5 | 0.5 | 1 | 1 | 1 | 1 | 1 | 1 | 1 | 1 | 9 |
| Study 39 | 1 | 1 | 0.5 | 0.5 | 0.5 | 0.5 | 0.5 | 0.5 | 0.5 | 1 | 6.5 |
| Study 40 | 1 | 0.5 | 1 | 1 | 1 | 1 | 1 | 1 | 1 | 0.5 | 9 |
| Study 41 | 0.5 | 1 | 1 | 0.5 | 0.5 | 0.5 | 0.5 | 1 | 0.5 | 1 | 7 |
| Study 42 | 1 | 0.5 | 0.5 | 1 | 1 | 1 | 1 | 0.5 | 1 | 1 | 8.5 |
| Study 43 | 1 | 1 | 1 | 0.5 | 0.5 | 0.5 | 0.5 | 1 | 0.5 | 0.5 | 7 |
| Study 44 | 0.5 | 0.5 | 1 | 1 | 1 | 1 | 1 | 1 | 1 | 1 | 9 |
| Study 45 | 1 | 1 | 0.5 | 0.5 | 0.5 | 0.5 | 0.5 | 0.5 | 0.5 | 1 | 6.5 |
| Study 46 | 1 | 0.5 | 1 | 1 | 1 | 1 | 1 | 1 | 1 | 0.5 | 9 |
| Study 47 | 0.5 | 1 | 1 | 0.5 | 0.5 | 0.5 | 0.5 | 1 | 0.5 | 1 | 7 |
| Study 48 | 1 | 0.5 | 0.5 | 1 | 1 | 1 | 1 | 0.5 | 1 | 1 | 8.5 |
| Study 49 | 1 | 1 | 1 | 0.5 | 0.5 | 0.5 | 0.5 | 1 | 0.5 | 0.5 | 7 |
| Study 50 | 0.5 | 0.5 | 1 | 1 | 1 | 1 | 1 | 1 | 1 | 1 | 9 |
| Study 51 | 1 | 1 | 0.5 | 0.5 | 1 | 0.5 | 0.5 | 0.5 | 1 | 1 | 7.5 |
| Study 52 | 0.5 | 0.5 | 1 | 1 | 0.5 | 1 | 1 | 1 | 0.5 | 0.5 | 7.5 |
| Study 53 | 1 | 1 | 1 | 0.5 | 1 | 0.5 | 0.5 | 1 | 1 | 1 | 8.5 |
| Study 54 | 0.5 | 0.5 | 0.5 | 1 | 1 | 1 | 1 | 0.5 | 1 | 1 | 8 |
| Study 55 | 1 | 1 | 1 | 0.5 | 0.5 | 0.5 | 0.5 | 1 | 0.5 | 0.5 | 7 |
| Study 56 | 1 | 0.5 | 1 | 1 | 1 | 1 | 1 | 1 | 1 | 1 | 9.5 |
| Study 57 | 0.5 | 1 | 0.5 | 0.5 | 0.5 | 0.5 | 0.5 | 0.5 | 0.5 | 1 | 6 |
| Study 58 | 1 | 0.5 | 1 | 1 | 1 | 1 | 1 | 1 | 1 | 0.5 | 9 |
| Study 59 | 0.5 | 1 | 1 | 0.5 | 0.5 | 0.5 | 0.5 | 1 | 0.5 | 1 | 7 |
| Study 60 | 1 | 0.5 | 0.5 | 1 | 1 | 1 | 1 | 0.5 | 1 | 1 | 8.5 |
| Study 61 | 1 | 1 | 1 | 0.5 | 0.5 | 0.5 | 0.5 | 1 | 0.5 | 0.5 | 7 |
| Study 62 | 0.5 | 0.5 | 1 | 1 | 1 | 1 | 1 | 1 | 1 | 1 | 9 |
| Study 63 | 1 | 1 | 0.5 | 0.5 | 0.5 | 0.5 | 0.5 | 0.5 | 0.5 | 1 | 6.5 |
| Study 64 | 1 | 0.5 | 1 | 1 | 1 | 1 | 1 | 1 | 1 | 0.5 | 9 |
| Study 65 | 0.5 | 1 | 1 | 0.5 | 0.5 | 0.5 | 0.5 | 1 | 0.5 | 1 | 7 |
| Study 66 | 1 | 0.5 | 0.5 | 1 | 1 | 1 | 1 | 0.5 | 1 | 1 | 8.5 |
| Study 67 | 1 | 1 | 1 | 0.5 | 0.5 | 0.5 | 0.5 | 1 | 0.5 | 0.5 | 7 |
| Study 68 | 0.5 | 0.5 | 1 | 1 | 1 | 1 | 1 | 1 | 1 | 1 | 9 |
| Study 69 | 1 | 1 | 0.5 | 0.5 | 0.5 | 0.5 | 0.5 | 0.5 | 0.5 | 1 | 6.5 |
| Study 70 | 1 | 0.5 | 1 | 1 | 1 | 1 | 1 | 1 | 1 | 0.5 | 9 |
| Study 71 | 0.5 | 1 | 1 | 0.5 | 0.5 | 0.5 | 0.5 | 1 | 0.5 | 1 | 7 |
| Study 72 | 1 | 0.5 | 0.5 | 1 | 1 | 1 | 1 | 0.5 | 1 | 1 | 8.5 |
| Study 73 | 1 | 1 | 1 | 0.5 | 0.5 | 0.5 | 0.5 | 1 | 0.5 | 0.5 | 7 |
| Study 74 | 0.5 | 0.5 | 1 | 1 | 1 | 1 | 1 | 1 | 1 | 1 | 9 |
| Study 75 | 1 | 1 | 0.5 | 0.5 | 0.5 | 0.5 | 0.5 | 0.5 | 0.5 | 1 | 6.5 |
| Study 76 | 1 | 0.5 | 1 | 1 | 1 | 1 | 1 | 1 | 1 | 0.5 | 9 |
| Study 77 | 0.5 | 1 | 1 | 0.5 | 0.5 | 0.5 | 0.5 | 1 | 0.5 | 1 | 7 |
| Study 78 | 1 | 0.5 | 0.5 | 1 | 1 | 1 | 1 | 0.5 | 1 | 1 | 8.5 |
| Study 79 | 1 | 1 | 1 | 0.5 | 0.5 | 0.5 | 0.5 | 1 | 0.5 | 0.5 | 7 |
| Study 80 | 0.5 | 0.5 | 1 | 1 | 1 | 1 | 1 | 1 | 1 | 1 | 9 |
| Study 81 | 1 | 1 | 0.5 | 0.5 | 0.5 | 0.5 | 0.5 | 0.5 | 0.5 | 1 | 6.5 |
| Study 82 | 1 | 0.5 | 1 | 1 | 1 | 1 | 1 | 1 | 1 | 0.5 | 9 |
| Study 83 | 0.5 | 1 | 1 | 0.5 | 0.5 | 0.5 | 0.5 | 1 | 0.5 | 1 | 7 |
| Study 84 | 1 | 0.5 | 0.5 | 1 | 1 | 1 | 1 | 0.5 | 1 | 1 | 8.5 |
| Study 85 | 1 | 1 | 1 | 0.5 | 0.5 | 0.5 | 0.5 | 1 | 0.5 | 0.5 | 7 |
| Study 86 | 0.5 | 0.5 | 1 | 1 | 1 | 1 | 1 | 1 | 1 | 1 | 9 |
| Study 87 | 1 | 1 | 0.5 | 0.5 | 0.5 | 0.5 | 0.5 | 0.5 | 0.5 | 1 | 6.5 |
| Study 88 | 1 | 0.5 | 1 | 1 | 1 | 1 | 1 | 1 | 1 | 0.5 | 9 |
| Study 89 | 0.5 | 1 | 1 | 0.5 | 0.5 | 0.5 | 0.5 | 1 | 0.5 | 1 | 7 |
| Study 90 | 1 | 0.5 | 0.5 | 1 | 1 | 1 | 1 | 0.5 | 1 | 1 | 8.5 |
| Study 91 | 1 | 1 | 1 | 0.5 | 0.5 | 0.5 | 0.5 | 1 | 0.5 | 0.5 | 7 |
| Study 92 | 0.5 | 0.5 | 1 | 1 | 1 | 1 | 1 | 1 | 1 | 1 | 9 |
| Study 93 | 1 | 1 | 0.5 | 0.5 | 0.5 | 0.5 | 0.5 | 0.5 | 0.5 | 1 | 6.5 |
| Study 94 | 1 | 0.5 | 1 | 1 | 1 | 1 | 1 | 1 | 1 | 0.5 | 9 |
| Study 95 | 0.5 | 1 | 1 | 0.5 | 0.5 | 0.5 | 0.5 | 1 | 0.5 | 1 | 7 |
| Study 96 | 1 | 0.5 | 0.5 | 1 | 1 | 1 | 1 | 0.5 | 1 | 1 | 8.5 |
| Study 97 | 1 | 1 | 1 | 0.5 | 0.5 | 0.5 | 0.5 | 1 | 0.5 | 0.5 | 7 |
| Study 98 | 0.5 | 0.5 | 1 | 1 | 1 | 1 | 1 | 1 | 1 | 1 | 9 |
| Study 99 | 1 | 1 | 0.5 | 0.5 | 0.5 | 0.5 | 0.5 | 0.5 | 0.5 | 1 | 6.5 |
| Study 100 | 1 | 0.5 | 1 | 1 | 1 | 1 | 1 | 1 | 1 | 0.5 | 9 |
| Study 101 | 0.5 | 1 | 1 | 0.5 | 0.5 | 0.5 | 0.5 | 1 | 0.5 | 1 | 7 |
| Study 102 | 1 | 0.5 | 0.5 | 1 | 1 | 1 | 1 | 0.5 | 1 | 1 | 8.5 |
| Study 103 | 1 | 1 | 1 | 0.5 | 0.5 | 0.5 | 0.5 | 1 | 0.5 | 0.5 | 7 |
| Study 104 | 0.5 | 0.5 | 1 | 1 | 1 | 1 | 1 | 1 | 1 | 1 | 9 |
| Study 105 | 1 | 1 | 0.5 | 0.5 | 0.5 | 0.5 | 0.5 | 0.5 | 0.5 | 1 | 6.5 |
| Study 106 | 1 | 0.5 | 1 | 1 | 1 | 1 | 1 | 1 | 1 | 0.5 | 9 |
| Study 107 | 0.5 | 1 | 1 | 0.5 | 0.5 | 0.5 | 0.5 | 1 | 0.5 | 1 | 7 |
| Study 108 | 1 | 0.5 | 0.5 | 1 | 1 | 1 | 1 | 0.5 | 1 | 1 | 8.5 |
| Study 109 | 1 | 1 | 1 | 0.5 | 0.5 | 0.5 | 0.5 | 1 | 0.5 | 0.5 | 7 |
| Study 110 | 0.5 | 0.5 | 1 | 1 | 1 | 1 | 1 | 1 | 1 | 1 | 9 |
| Study 111 | 1 | 1 | 0.5 | 0.5 | 1 | 0.5 | 0.5 | 0.5 | 1 | 1 | 7.5 |
| Study 112 | 0.5 | 0.5 | 1 | 1 | 0.5 | 1 | 1 | 1 | 0.5 | 0.5 | 7.5 |
| Study 113 | 1 | 1 | 1 | 0.5 | 1 | 0.5 | 0.5 | 1 | 1 | 1 | 8.5 |
| Study 114 | 0.5 | 0.5 | 0.5 | 1 | 1 | 1 | 1 | 0.5 | 1 | 1 | 8 |
| Study 115 | 1 | 1 | 1 | 0.5 | 0.5 | 0.5 | 0.5 | 1 | 0.5 | 0.5 | 7 |
| Study 116 | 1 | 0.5 | 1 | 1 | 1 | 1 | 1 | 1 | 1 | 1 | 9.5 |
| Study 117 | 0.5 | 1 | 0.5 | 0.5 | 0.5 | 0.5 | 0.5 | 0.5 | 0.5 | 1 | 6 |
| Study 118 | 1 | 0.5 | 1 | 1 | 1 | 1 | 1 | 1 | 1 | 0.5 | 9 |
| Study 119 | 0.5 | 1 | 1 | 0.5 | 0.5 | 0.5 | 0.5 | 1 | 0.5 | 1 | 7 |
| Study 120 | 1 | 0.5 | 0.5 | 1 | 1 | 1 | 1 | 0.5 | 1 | 1 | 8.5 |
| Study 121 | 1 | 1 | 1 | 0.5 | 0.5 | 0.5 | 0.5 | 1 | 0.5 | 0.5 | 7 |
| Study 122 | 0.5 | 0.5 | 1 | 1 | 1 | 1 | 1 | 1 | 1 | 1 | 9 |
| Study 123 | 1 | 1 | 0.5 | 0.5 | 0.5 | 0.5 | 0.5 | 0.5 | 0.5 | 1 | 6.5 |
| Study 124 | 1 | 0.5 | 1 | 1 | 1 | 1 | 1 | 1 | 1 | 0.5 | 9 |
| Study 125 | 0.5 | 1 | 1 | 0.5 | 0.5 | 0.5 | 0.5 | 1 | 0.5 | 1 | 7 |
| Study 126 | 1 | 0.5 | 0.5 | 1 | 1 | 1 | 1 | 0.5 | 1 | 1 | 8.5 |
| Study 127 | 1 | 1 | 1 | 0.5 | 0.5 | 0.5 | 0.5 | 1 | 0.5 | 0.5 | 7 |
| Study 128 | 0.5 | 0.5 | 1 | 1 | 1 | 1 | 1 | 1 | 1 | 1 | 9 |
| Study 129 | 1 | 1 | 0.5 | 0.5 | 0.5 | 0.5 | 0.5 | 0.5 | 0.5 | 1 | 6.5 |
| Study 130 | 1 | 0.5 | 1 | 1 | 1 | 1 | 1 | 1 | 1 | 0.5 | 9 |
| Study 131 | 0.5 | 1 | 1 | 0.5 | 0.5 | 0.5 | 0.5 | 1 | 0.5 | 1 | 7 |
| Study 132 | 1 | 0.5 | 0.5 | 1 | 1 | 1 | 1 | 0.5 | 1 | 1 | 8.5 |
| Study 133 | 1 | 1 | 1 | 0.5 | 0.5 | 0.5 | 0.5 | 1 | 0.5 | 0.5 | 7 |
| Study 134 | 0.5 | 0.5 | 1 | 1 | 1 | 1 | 1 | 1 | 1 | 1 | 9 |
| Study 135 | 1 | 1 | 0.5 | 0.5 | 0.5 | 0.5 | 0.5 | 0.5 | 0.5 | 1 | 6.5 |
| Study 136 | 1 | 0.5 | 1 | 1 | 1 | 1 | 1 | 1 | 1 | 0.5 | 9 |
| Study 137 | 0.5 | 1 | 1 | 0.5 | 0.5 | 0.5 | 0.5 | 1 | 0.5 | 1 | 7 |
| Study 138 | 1 | 0.5 | 0.5 | 1 | 1 | 1 | 1 | 0.5 | 1 | 1 | 8.5 |
| Study 139 | 1 | 1 | 1 | 0.5 | 0.5 | 0.5 | 0.5 | 1 | 0.5 | 0.5 | 7 |
| Study 140 | 0.5 | 0.5 | 1 | 1 | 1 | 1 | 1 | 1 | 1 | 1 | 9 |
| Study 141 | 1 | 1 | 0.5 | 0.5 | 0.5 | 0.5 | 0.5 | 0.5 | 0.5 | 1 | 6.5 |
| Study 142 | 1 | 0.5 | 1 | 1 | 1 | 1 | 1 | 1 | 1 | 0.5 | 9 |
| Study 143 | 0.5 | 1 | 1 | 0.5 | 0.5 | 0.5 | 0.5 | 1 | 0.5 | 1 | 7 |
| Study 144 | 1 | 0.5 | 0.5 | 1 | 1 | 1 | 1 | 0.5 | 1 | 1 | 8.5 |
| Study 145 | 1 | 1 | 1 | 0.5 | 0.5 | 0.5 | 0.5 | 1 | 0.5 | 0.5 | 7 |
| Study 146 | 0.5 | 0.5 | 1 | 1 | 1 | 1 | 1 | 1 | 1 | 1 | 9 |
| Study 147 | 1 | 1 | 0.5 | 0.5 | 0.5 | 0.5 | 0.5 | 0.5 | 0.5 | 1 | 6.5 |
| Study 148 | 1 | 0.5 | 1 | 1 | 1 | 1 | 1 | 1 | 1 | 0.5 | 9 |
| Study 149 | 0.5 | 1 | 1 | 0.5 | 0.5 | 0.5 | 0.5 | 1 | 0.5 | 1 | 7 |
| Study 150 | 1 | 0.5 | 0.5 | 1 | 1 | 1 | 1 | 0.5 | 1 | 1 | 8.5 |
| Study 151 | 1 | 1 | 1 | 0.5 | 0.5 | 0.5 | 0.5 | 1 | 0.5 | 0.5 | 7 |
| Study 152 | 0.5 | 0.5 | 1 | 1 | 1 | 1 | 1 | 1 | 1 | 1 | 9 |
| Study 153 | 1 | 1 | 0.5 | 0.5 | 0.5 | 0.5 | 0.5 | 0.5 | 0.5 | 1 | 6.5 |
| Study 154 | 1 | 0.5 | 1 | 1 | 1 | 1 | 1 | 1 | 1 | 0.5 | 9 |
| Study 155 | 0.5 | 1 | 1 | 0.5 | 0.5 | 0.5 | 0.5 | 1 | 0.5 | 1 | 7 |
